# Italian Adaptation of the Revised Multicultural Ideology Scale (MCI‐r)

**DOI:** 10.1002/ijop.70073

**Published:** 2025-07-07

**Authors:** Chiara Parisse, Stefano Livi, Laura Prislei, Mara Marini, John W. Berry

**Affiliations:** ^1^ Department of Psychology of Development and Socialization Processes Sapienza University of Rome Italy; ^2^ Department of Neuroscience, Imaging and Clinical Sciences University of Chieti‐Pescara Chieti Italy; ^3^ Queen's University Kingston Canada

**Keywords:** intergroup relationship, multicultural ideology, political orientations, psychometrics proprieties

## Abstract

The rise of migrants from different cultural backgrounds in Italy highlights the need to promote harmonious coexistence between them and the local population. A key factor in addressing this challenge is the level of acceptance in society of a multicultural ideology. An instrument to measure this concept has recently been revised into the Revised Multicultural Ideology Scale (MCI‐r). Despite the urgency of adopting this broader ideology in Italy, no adaptation of the current scale has been made in the Italian context. To bridge this gap, our studies aim to adapt the MCI‐r scale to the Italian context and assess its predictive validity on variables crucial for positive intergroup relations. We collected data from two distinct samples: one from Prolific (*N* = 301) and another from Sapienza University (*N* = 204). Using confirmatory factor analysis, measurement invariance, and convergent and discriminant validity analyses, we investigated the psychometric properties of the scale based on its recent validations in other societies. Furthermore, we tested its predictive validity concerning the quality of contact with migrants and political orientation. Our findings supported a four‐factor solution and a higher‐order dimension. Additionally, results supported the predictive validity of MCI‐r and the superordinate dimension on positive contact with migrants and political orientations.

In 2022, Italy recorded an increased arrival of long‐term new migrants with different cultural backgrounds, positioning Italy among the European countries with the highest increases in migrant arrivals (OECD 2023). Moreover, the XIII Annual Report of the Italian Ministry of Labour and Social Policies (Italian Ministry of Labour and Social Policies 2023) identified ethnic discrimination as the primary barrier to migrant integration in Italy. The rise of multiculturalism in Italy (OECD 2023), combined with persistent ethnic discrimination (Italian Ministry of Labour and Social Policies 2023), highlights the need to provide tools to understand and manage intercultural relations. As a response to this need, the revised version of the Multicultural Ideology Scale (MCI‐r) was introduced as a valuable conceptual framework by Stogianni et al. (2023).

Given Italy's growing cultural diversity and the original goal set by Stogianni et al. (2023) of providing a cross‐cultural validation tool, we aimed to adapt and evaluate the psychometric properties of the MCI‐r scale in the Italian context through two studies. Specifically, we adapted the MCI‐r scale and assessed its psychometric properties in the first study. In the second one, we sought to further validate our findings by confirming the factor structure in a second independent sample and testing the scale's predictive validity in relation to key variables in intergroup relations—namely, the quality of contact with migrants and political orientation.

## 1 Multiculturalism

Intercultural relations have long been a significant topic in various national contexts (Sam and Berry 2006). Consequently, cultural psychology has developed research lines to explore how cultures in contact can positively coexist within a country (Sam and Berry 2006). This field of research led to the development of acculturation strategies (Sam and Berry 2006). These strategies focused on understanding how migrants seek to relate to the host society (their acculturation strategies) and how the local population expects incoming migrants to acculturate (their acculturation expectations). These reciprocal processes between migrants and host populations have sparked interest in broader societal attitudes toward cultural diversity, paving the way for the conceptualisation of Multicultural Ideology.

A first study of the concept of Multicultural Ideology was carried out by Berry et al. (1977) using samples from the general population, as well as immigrant and ethnic group members. Most later studies mainly focused on migrants' acculturation strategies and adaptation (see Sam and Berry 2006). However, more recently, the literature has increasingly highlighted the bidirectional nature of effective cultural adaptation, examining the sociopsychological factors that shape the local population's acceptance of immigrants (e.g., Prislei et al. 2024) and investigating acculturation processes from the perspective of the local population as well (e.g., Sam and Berry 2006; see also Zagefka et al. 2023). The conceptualisation of Multicultural Ideology has its roots in the diverse theoretical definitions of the broader multiculturalism.

Multiculturalism has been defined in three ways (e.g., Berry et al. 1977). The first definition relates to the demographic characteristics of a given area, describing the ethnic composition of a society. The second pertains to policies, including governmental strategies to manage cultural diversity within a specific territory^1^. Finally, multiculturalism has been defined in psychological terms as a political ideology that supports and promotes the acceptance of cultural diversity, the maintenance of cultural identities, and the social participation of all people in the larger society (Berry and Kalin 1995). Within this definition, the ideological dimension of multiculturalism emerged as a framework for understanding how positive intercultural relations can be enhanced (Berry et al. 1977). This construct reflects an inclusive view of diversity, asserting that a society consists of different ethnocultural groups with equal rights, regardless of their size or power (Berry et al. 1977). In this sense, cultural diversity is not merely viewed as a demographic characteristic or a policy; it is seen as a global evaluation by citizens of migrants and ethnocultural groups, playing a significant role in the positive evolution of intercultural relations in heterogeneous societies (Berry and Kalin 1995). Specifically, multicultural ideology refers to public attitudes toward the acceptance or rejection of diversity, as well as the support for policies that promote immigrant integration and inclusion (Berry and Ward 2016). It posits that positive intergroup relations are achievable when societies genuinely accept cultural diversity. The presence of a multicultural ideology within a society can reduce discrimination and prejudice by fostering positive relations between migrants and natives (Berry and Kalin 1995). Furthermore, multicultural ideology may be a crucial precondition for fostering integration in a multicultural society (Berry and Kalin 1995). Considering this, understanding the relationship between acculturation strategies and multicultural ideology becomes crucial, as they are deeply intertwined in shaping the dynamics of integration within multicultural societies.

The relationship between acculturation strategies and multicultural ideology is particularly strong, especially in the early conceptualisation of this construct. Specifically, the first MCI scale (Berry et al. 1977) was based on the same two dimensions as the acculturation strategies framework (cultural maintenance and contact/participation). However, while these concepts are closely related, they remain distinct. Multicultural ideology is a more stable and overarching belief system, whereas acculturation strategies tend to be more variable and context‐dependent. The relationship between acculturation preferences and multicultural ideology remains unclear, as only one longitudinal study has investigated the direction of this association. In that study, acculturation preferences predicted multicultural ideology (Lefringhausen et al. 2024). Cross‐sectional studies, instead, have shown that multicultural ideology can shape acculturation preferences (e.g., Sam and Berry 2006), as is typically the case with ideological variables^2^. However, unlike other ideologies, multicultural ideology is best understood as a general attitude grounded in an inclusive and egalitarian perspective. Specific contextual factors can shape it, such as acculturation strategies (Lefringhausen et al. 2024) and contact quality (Parisse et al. 2025). However, it can also be considered relatively stable over time, fostering context‐specific variables such as integration behaviours (Parisse et al. 2025).

Over time, the concept of multicultural ideology has evolved and has been understood in various ways. Initially, support for multiculturalism was primarily conceptualised through the lens of acculturation strategies (Berry et al. 1977). These strategies provided a framework to explore how migrants and the larger society interact and adjust to cultural diversity. However, as the understanding of multiculturalism grew, the scope of this conceptualisation expanded to include more policy‐oriented dimensions (Rex 1998) and the expectations of the majority population regarding migrant acculturation (see Sam and Berry 2006). At the same time, these expectations incorporated the importance of respecting and accepting the cultural distinctiveness of migrants (see Zagefka et al. 2023).

Despite differences in their approaches, these early conceptualisations shared core principles, emphasising the fundamental elements of a pluralistic society, which included addressing the needs and roles of both migrant minorities and the local majorities, as well as considering both private and public policies and the normative expectations of how cultures should coexist (see Sam and Berry 2006). More recently, other ideologies have been proposed (see Grigoryev and Berry 2021) that seek to address these same issues.

Building on these ideas, various tools were developed over time to assess multicultural ideology tailored to different societal contexts. It has been examined through both qualitative and quantitative approaches. However, quantitative tools have become more prominent over time due to their broader applicability and ability to assess larger populations^3^. In this context, Berry et al. (1977) and Berry and Kalin (1995) developed the first MCI scale, which aims to understand how individuals believe ethnocultural groups should relate to the broader society. The MCI scale, which included 10 items, was first used with a large sample of Canadians. The scale assessed key ideologies related to assimilation, segregation, and integration while addressing how diversity might impact national unity. Despite its contributions, the MCI did not fully capture all aspects of multicultural ideology.

As a result, a revised version of the scale, the Multicultural Attitude Scale (MAS), was developed by Breugelmans and Van De Vijver (2004). The MAS included 28 items that covered a broader range of topics, including attitudes toward diversity, the acculturation strategies employed by both minority and majority groups, and the general attitudes toward equal participation and interaction between immigrants and mainstream society.

With the increasing cultural differences and complexity of societies characterised by individuals from diverse ethnic backgrounds and the persistent discriminatory behaviours faced by these minorities (see Grigoryev et al. 2020), multicultural ideology remains crucial. It remains a key factor in striving for harmonious coexistence between intercultural groups. Given these cultural contexts, it has become evident that modifications to the current scales are necessary to capture and address the current societies' dynamics.

## 2 Multicultural Ideology Scale—Revised

To assess multicultural ideology in increasingly culturally diverse societies, a revised version of the MCI‐r was developed by Stogianni et al. (2023). The previous assessment tools identified key dimensions related to the value of cultural maintenance, diversity, social interaction, and equity between local populations and migrants, which are core aspects of multicultural ideology. However, these tools fell short of fully addressing all these dimensions and neglected other relevant factors crucial to understanding this ideology in modern multicultural societies.

To address this more complex multicultural reality, Stogianni et al. (2023) expanded on the general conceptualisation of multicultural ideology, introducing new dimensions that were not covered in the earlier version of the MCI scale while also incorporating some dimensions previously explored by other researchers.

The MCI‐r scale comprises six subscales covering three conceptual attitude dimensions. One of these dimensions, Cultural Maintenance, is carried over from the earlier version (i.e., Berry et al. 1977) and focuses on integrating immigrant groups. The other two dimensions are new and address the impact of cultural diversity and managing cross‐cultural differences between groups. The three subscales—Cultural Maintenance (CM), Equity/Inclusion (EQ), and Social Interaction (SI)—reflect the integration strategy, emphasising the right of ethnic minority groups to preserve their cultural heritage, the social participation of all cultural groups, and the interactions between majority and minority group members in various contexts. Although the previous version of the MCI scale (i.e., Berry et al. 1977) partially addressed the dimensions of EQ and SI, it included only a few items, with the MAS scale (Breugelmans and Van De Vijver 2004) only partially covering the EQ dimension. The MCI‐r scale, however, fully operationalised these dimensions.

In addition to these three subscales, three new ones were introduced: The Extent of Differences (DI), Consequences of Diversity (CD), and Essentialistic Boundaries (EB). The DI measures the perception of cultural differences between groups, EB examines beliefs about the distinctiveness of ethnic group members, and CD explores conflictual relations between intercultural groups.

These dimensions are considered valuable enhancements to the previous measure, as research has shown their significant associations with acculturation preferences and attitudes toward migrants (see Stogianni et al. 2023; Lefringhausen et al. 2024).

Stogianni et al. (2023), Lefringhausen et al. (2024), and Chahar Mahali et al. (2025) examined the psychometric properties of the MCI‐r scale across four different samples: the British, German, Luxembourgish, and Canadian populations. The first two studies only retained support for the four‐factor solution, which included the dimensions of CM, EQ, SI, and CD.

Support for the six‐factor solution was found only in the psychometric evaluation of the scale conducted by Chahar Mahali et al. (2025). However, the authors noted that the four‐factor model provided a significantly better fit than the six‐factor one. They also highlighted the lack of association between the DI dimension and the other dimensions, as well as the negative association between EB and the others, suggesting, along with Stogianni et al. (2023), that these two dimensions might require revision or could be irrelevant to multicultural ideology in the context under study. Additionally, Stogianni et al. (2023) identified different interpretations of some scale items between the German and Luxembourgish samples, highlighting the need to develop cross‐cultural tools. Therefore, adapting this new scale to various cultural contexts is essential (Lefringhausen et al. 2024).

In this way, the present studies aim to adapt the MCI‐r scale for native‐born Italian samples by exploring the most suitable factor solution for the Italian context, based on comparison models from the original validation, and to contribute to the cross‐cultural validation of the measure. This study also offers a pertinent tool for investigating intergroup relations in Italy.

To achieve our aims, we conducted two studies. The first one involved testing a Confirmatory Factor Analysis (CFA), assessing convergent validity (using Average Variance Extracted, AVE), discriminant validity (using the Heterotrait‐Monotrait Ratio of Correlations, HTMT), and measurement invariance across genders for the most appropriate factor solution. In the second one, we retested the factor solution identified in Study 1 to corroborate our findings further. Lastly, we evaluated the scale's predictive validity by conducting correlation analyses with the quality of contact with migrants and political orientation, as previously done by Lefringhausen et al. (2024).

We decided to conduct two studies to strengthen the validity of our findings by examining the factor structure across two independent native‐born Italian samples, each obtained using different sampling methods. This approach allows us to assess the consistency of the factor structure within the Italian context.

## 3 Method

### 3.1 Participants of Study 1 and 2

We gathered data in 2023 from 301 native‐born Italians recruited through Prolific for Study 1 and 218 students from Sapienza University of Rome for Study 2. The studies obtained ethical approval^4^.

The participants completed the survey online using Qualtrics. Participants of Study 1 received compensation of £16.20 per hour. In both studies, participants were initially provided with demographic information. Subsequently, the MCI‐r items were presented, and only in Study 2 were the participants presented with contact quality and political orientation scales.

Considering the inclusion criteria provided by Stogianni et al. (2023) we included only participants born in Italy who spoke Italian.

In Study 1, when testing measurement invariance across gender, we excluded participants who identified as ‘non‐binary’ due to the small sample size (i.e., 6). The sample for performing CFA, convergent and discriminant validity comprised 301 participants whose attention was assessed using a single item. The sample for Study 2 resulted in a final sample of 204 participants whose attention was also assessed.

The demographics are summarised in Table 1.

**TABLE 1:** Demographics of the Study 1 and 2.

	Study 1		Study 2	
Demographics	N%		N%	
Age				
*M*(SD)	29.9		20.5	
	(4.22)		(1.98)	
Gender				
Woman	147	48.8	129	63.2
Man	148	49.2	71	34.8
Not binary	6	1.9	3	1.5
I prefer not to disclose	—	—	1	0.5
Language spoken at home				
Italian	290	96.4	194	95.1
Italian and another one	11	3.7	10	4.9
Education				
Compulsory education (until 16 years old)	1	0.3	—	—
High School Diploma	88	29.2	199	97.5
Bachelor's degree	95	31.6	3	1.5
Master's degree	102	33.9	2	1
PhD	15	4.9	—	—
Migration background				
None	286	95.0	183	89.71
One parent was born abroad	14	4.7	1	0.49
Two parents were born abroad	1	0.3	—	—

### 3.2 Study 1

The first study aims to contribute to the cross‐cultural validation of the MCI‐r, providing a valuable tool to address the challenges posed by the evolving migration landscape in Italy. To achieve this, we aim to test all the factor solutions proposed in previous studies (e.g., Stogianni et al. 2023) within a native‐born Italian sample. For testing the most suitable factor solution within the Italian context, we conducted the following CFA based on previous study by Stogianni et al. (2023): the originally conceptualised six‐factor model; a four‐factor model, excluding EB and DI, only whether these dimensions would not correlate with the other factors; a one‐factor model; a hierarchical factor model assuming a superordinate latent factor Multicultural Ideology (MI) encompassing the four dimensions; a bifactor model to assess the suitability of a structure that simultaneously accounts for both unidimensional and multidimensional properties.

Secondly, we analyse the scale's psychometric properties in Italy, assessing its convergent and discriminant validity using the AVE and the HTMT^5^. Stogianni et al. (2023) previously employed both methods to assess the same psychometric properties.

Lastly, we conducted Multigroup Confirmatory Factor Analysis (MG‐CFA) across genders (as previously examined by Stogianni et al. 2023), as examining gender differences in multicultural ideology is particularly relevant given the conflicting findings in the literature. Some studies suggest that men exhibit higher levels of prejudice against migrants and lower awareness of social injustice (Navarrete et al. 2010), while others have found no significant gender differences in multicultural attitudes (e.g., Stogianni et al. 2023). Given these mixed findings, we consider it essential to explore the potential effects of gender on multicultural ideology within the Italian context as well.

### 3.3 Measures of Study 1

The MCI‐r scale consisted of 24 items, initially translated using the back translation procedure by Brislin (2000)^6^. The items, some reversed (see Table A1), were rated on a 5‐point Likert scale (1 = strongly disagree to 5 = strongly agree). The scale investigated people's attitudes across six dimensions: CM, SI, EQ, DI, CD, and EB (see Appendix).

#### 3.3.1 *Analyses of Study 1* ^7^

We conducted a preliminary check to assess whether the distribution of the variables was normal. The skewness and kurtosis indices were examined to ensure that the data met the assumptions for proceeding with CFA (Tabachnick and Fidell 2013). Only the EQ dimension did not have a normal distribution. Then we conducted CFA using a robust estimator as previously done by Stogianni et al. (2023).

We conducted a CFA using R studio (R Core Team 2021) with a maximum likelihood robust (MLM) estimator.

Model fit was assessed using the following indices: incremental or comparative indices (TLI, CFI), approximation indices (RMSEA), and sample indices of fit (SRMR)^8^. Additionally, we used CFI differences to identify the best model among all those tested^9^. Subsequently, we conducted convergent and discriminant validity tests for the factor solution that emerged from CFA, using the AVE and the HTMT, respectively^10^. Finally, we employed MG‐CFA to examine measurement invariance across gender. MG‐CFA is the standard method for investigating the extent to which measures remain invariant across genders, as Stogianni et al. (2023) suggested. We assessed three types of invariance: configural, metric, and scalar. Model comparisons were conducted using the Chi‐square difference test (Byrne et al. 1989) and the CFI test (Cheung and Rensvold 2002), given that the former is sensitive to sample size.

### 3.4 Measurement Model of Study 1

In the first step, we evaluated the six‐factor model with correlations between factors, aligning with the initial validation proposed by Stogianni et al. (2023). However, the six‐factor model demonstrated poor fit indices and showed no associations between the DI and EB dimensions and the other factors. Given the lack of correlation between DI and EB with the remaining dimensions of the scale, as also observed by Lefringhausen et al. (2024) and Stogianni et al. (2023), we tested the four‐factor solution, dropping DI and EB.

The four‐factor model demonstrated good fit indices, excluding the DI and EB dimensions^11^. Subsequently, we tested a one‐factor solution to rule out the possibility that a nested model might better explain the data. However, this model exhibited poor fit indices in its full version and when DI and EB were removed.

Comparisons of CFI differences confirmed that the four‐factor solution, excluding DI and EB, was the best‐fitting model. We further examined the correlations between the factors through correlation analysis, summarised in Table 3, which revealed positive associations. This suggested the presence of a potential second‐order latent factor encompassing the four sub‐dimensions. Based on this assumption, we tested a hierarchical model. The second‐order four‐factor model, excluding DI and EB, also demonstrated good fit indices.

Only marginal differences emerged between the four‐factor solution and the second‐order model. Consequently, the scale can be used by considering the four separate dimensions or as a composite index calculated as the mean score of the four dimensions (i.e., MI). However, for the subsequent analyses (convergent validity, discriminant validity, and measurement invariance), we retained the simpler model, that is, the four‐factor solution. Finally, we explored bifactor models to assess both unidimensional and multidimensional structures. However, no bifactor models—whether including or excluding DI and EB—yielded convergence.

All the factor solution model fit indices were reported in Table 2, excluding the bifactor models that do not reach convergence. Additionally, we assessed the reliability of the factors emerging from the four‐factor and hierarchical models using omega (*ω*) estimates, which indicated good reliability for each dimension and the composite scale (see Table 3).

**TABLE 2:** Summarised model fits (in bold the best fitting model) of Study 1.

Model	*χ* ^2^(df) scaled	CFI scaled	TLI scaled	RMSEA Scaled [90% CI]	SRMR scaled	ΔCFI scaled
Four‐factor model with EB and DI dropped	**240.518 (98)**	**0.924**	**0.907**	**0.074 (0.062, 0.086)**	**0.051**	**—**
Second order four‐factor model with DI and EB dropped	**245.377 (100)**	**0.922**	**0.907**	**0.074 (0.062, 0.086)**	**0.52**	**0.002**
One‐factor model	1401.942 (252)	0.571	0.530	0.131 (0.125, 138)	0.117	0.353
One‐factor model with EB and DI dropped	489.349 (104)	0.787	0.755	0.120 (0.110, 0.131)	0.077	0.137
Six‐factor model with correlations between factors	599.408 (237)	0.867	0.845	0.075 (0.068, 0.083)	0.101	0.057

Abbreviations: ΔCFI, Comparative fit index differences; *χ* ^2^(df), Chi‐square (degree of freedom); CFI, Comparative fit index; RMSEA, Root‐mean‐square error of approximation; SRMR, Standardised root mean square residual; TLI, Tucker–Lewis index.

**TABLE 3:** Correlations between dimensions of the four‐factor model and reliability coefficients of Study 1.

	*M*	SD	1	2	3	4	*ω*
1. CM	3.85	0.78	—	—	—	—	0.82
2. EQ	4.49	0.72	0.46***	—	—	—	0.88
3. SI	4.00	0.75	0.66***	0.58***	—	—	0.77
4. CD	3.60	0.85	0.66***	0.57***	0.71***	—	0.74
5. MI	3.98	0.65	—	—	—	—	0.88

*Note*: We did not investigate the correlation between the MI dimension and the others as it was composed of their mean scores.

*** *p* < 0.001.

### 3.5 Convergent and Discriminant Validity of Study 1

We assessed convergent validity using the AVE for the dimensions of the four‐factor model (Cheung and Wang 2017).

In our findings, we obtained support for convergent validity for CM (AVE = 0.51) and EQ (AVE = 0.55) but not for SI (AVE = 0.41) and CD (AVE = 0.48).

Subsequently, we examined discriminant validity using the HTMT for the same dimension of the four‐factor solution (Cheung and Wang 2017).

Our results indicated discriminant validity for CM and EQ dimensions. A summary of discriminant validity is presented in Table 4.

**TABLE 4:** Discriminant validity using HTMT of Study 1.

	1	2	3	4
1. CM	—	—	—	—
2. EQ	0.555	—	—	—
3. SI	0.834	0.736	—	—
4. CD	0.829	0.712	0.939	—

### 3.6 Measurement Invariance Across Gender of Study 1

The four‐factor solution proved to be the superior model, leading us to conduct measurement invariance across genders for this model. We examined three types of invariances (configural, metric, and scalar).

The results supported all three invariances^12^, showing the absence of differences between males and females, and the summarised findings are presented in Table 5.

**TABLE 5:** Measurement invariance.

Model	*χ* ^2^	df *χ* ^2^	Δ df *χ* ^2^	*p* (Δ *χ* ^2^)	CFI	ΔCFI
Configural invariance	359.863	196			0.921	
Metric invariance	384.56	208	20.650	0.05574	0.915	0.006
Scalar invariance	394.63	220	11.338	0.50018	0.916	0.005

Abbreviations: *χ* ^2^, Chi‐square; Δ df *χ* ^2^, Chi‐square degree of freedom differences; ΔCFI, Comparative fit index differences; CFI, Comparative fit index; df *χ* ^2^, Chi‐square degree of freedom; *p* (Δ df *χ* ^2^), Chi‐square degree of freedom differences *p*.

## 4 Study 2

The primary aim of the second study is to corroborate further the four‐factor solution and the hierarchical structure that emerged from Study 1. In addition, this study seeks to investigate the predictive validity of the MCI‐r scale in relation to political orientations and the quality of intergroup contact. Previous research has established a connection between multicultural attitudes, political orientations, and the quality of intergroup contact (Rydgren 2007).

As Rydgren (2007) highlighted, sociological studies have delineated the key distinctions between right‐wing and left‐wing political orientations in contemporary societies, particularly concerning ethno‐pluralism. The researcher stated that right‐wing political ideologies tend to emphasise the preservation of national identity by advocating for the separation of different ethnic groups. In contrast, left‐wing orientations are more closely aligned with multicultural ideologies, emphasising the importance of preserving migrants' cultural heritage. Despite these theoretical discussions, the relationship between multicultural attitudes and political orientation remains underexplored in the literature. Most prior studies have approached multiculturalism as a policy framework or a demographic characteristic (e.g., Rex 1998). In contrast, the present study investigates the association between multiculturalism as an attitudinal construct and political orientation.

The existing literature on the relationship between the quality of intergroup contact and multicultural ideology is relatively sparse. Lefringhausen et al. (2024) conducted correlational analyses to examine this relationship within the framework of convergent validity; however, their study primarily focused on the frequency of intergroup contact rather than its qualitative aspects. Based on this finding, in the present study, we chose to focus exclusively on the quality of intergroup contact. This dimension has been extensively emphasised in prior research as a crucial factor in fostering positive intergroup relations, especially among majorities and in Italy (Vezzali et al. 2023).

Given these findings and the relative scarcity of studies directly examining these associations, the present study aims to explore the relationship between multicultural ideology—derived from a previous CFA—and political orientation and the quality of intergroup contact through correlational analysis. Specifically, we examined the associations between each identified dimension of multicultural ideology, including the hierarchical dimension and the outcomes of interest. Based on existing theoretical and empirical evidence, we hypothesised that all dimensions of the revised MCI and the hierarchical factor would negatively correlate with right‐wing political orientation. Furthermore, we hypothesised that all scale dimensions and the hierarchical factor would positively correlate with the quality of intergroup contact toward migrants.

### 4.1 Measures of Study 2

#### 4.1.1 The MCI‐r Scale

We used the same scale as in Study 1, considering only the four dimensions that emerged from the CFA conducted previously^13^.

#### 4.1.2 Political Orientation (PO)^14^

We measured participants' political orientation with 1 item: “Some people talk about right, left, or centre to discuss political parties and personalities. Given this distinction, where would you place yourself from 1 (Extreme left) to 10 (Extreme right).”

#### 4.1.3 Quality of Contact (QC)

We measured the quality of contact with migrants by asking participants to rate their contact with migrants using four semantic differential items (e.g., hostile/friendly) used in previous studies (e.g., Capozza et al. 2013). The scale was on a 5‐point scale (1 indicated the negative and 5 the positive pole).

### 4.2 Predictive Validity^15^

We conducted the predictive validity test by performing a correlation analysis between the hierarchical dimension of multicultural ideology, each sub‐dimension, and both the quality of contact with migrants and political orientation.

We conducted a preliminary check to assess whether the distribution was normal. The skewness and kurtosis indices were adequate except for the EQ dimension (Tabachnick and Fidell 2013). For this reason, we performed a correlation analysis using Spearman.

The association between variables was summarised in Table 6.

**TABLE 6:** Predict validity bivarcorrelations of Study 2.

	*M*	SD	1	2	3	4	5	6	7	*ω*
1. MI	4.07	0.54	—							**0.81**
2. CM	4.06	0.65	0.75***	—						**0.72**
3. EQ	4.6	0.63	0.69***	0.40***	—					**0.88**
4. SI	4.04	0.65	0.83***	0.52***	0.49***	—				**0.64**
5. CD	3.60	0.75	0.84***	0.48***	0.47***	0.61***	—			**0.71**
6. QC	3.79	0.74	0.56***	0.46***	0.40***	0.51***	0.44***	—		**0.85**
7. PO	4.47	1.52	−0.41***	−0.37***	−0.23***	−0.33***	−0.36***	−0.19***	—	**—**

*** *p* < 0.001.

## 5 Discussion

Multicultural ideology is crucial in promoting positive relations between different cultural groups. However, a reliable and contextually appropriate measurement tool is essential to achieve positive intergroup relations in the cultural Italian landscape.

For this reason, the present study aims to adapt the MCI‐r scale within the Italian context. Additionally, the study aims to contribute to the broader cross‐cultural adaptation of the proposed scale.

The first study's findings confirmed the four‐factor solution (dropping DI and EB), which aligns with prior cross‐cultural validations of the MCI‐r (e.g., Lefringhausen et al. 2024). This suggests that these two sub‐scales may require further refinement in future research or are irrelevant within the Italian context and in the other countries where they were excluded.

Comparative analyses with alternative models addressed the presence of a superordinate dimension underlying multicultural ideology. This overarching construct encompasses the four validated sub‐dimensions, supporting the notion that multicultural ideology is a broad framework shaping intergroup relations, aligning with the original validation of the measure (e.g., Stogianni et al. 2023).

Additionally, an examination of convergent and discriminant validity partially confirmed the scale's effectiveness, particularly in the CM and EQ dimensions. These results suggest that while the CM and EQ dimensions are well‐defined and distinct, the SI and CD dimensions may require further refinement. The weaker convergent validity of SI and CD suggests that their measurement items may not fully capture their intended constructs, possibly due to ambiguity in item wording or conceptual overlap with other dimensions. In this way, results are inconsistent, as Stogianni et al. (2023) found different convergent and discriminant validity across the two samples employing the same tests. This may suggest that the four dimensions may vary slightly across cultural contexts regarding convergent and discriminant validity.

Lastly, the study established measurement invariance across genders, which aligns with previous findings by Stogianni et al. (2023), allowing for meaningful comparisons between males and females in their multicultural attitudes—a dimension that has been relatively underexplored in previous research.

The second study explores the predictive validity of multicultural ideology by examining its relationship with political orientation and intergroup interactions. The findings indicate that a high multicultural ideology is associated with more positive intergroup interactions, specifically in the form of positive contact with migrants. Likewise, a strong multicultural ideology is negatively associated with right‐wing political orientations, aligning with previous research (Rydgren 2007) and the recent study by Lefringhausen et al. (2024). When examining correlations with each sub‐dimension, all sub‐dimensions show significant associations with the quality of contact with migrants. However, SI emerges as the most strongly related dimension. This aligns with Lefringhausen et al.'s (2024) findings, which suggest that attitudes toward social integration play a crucial role in shaping positive contact with migrants. Nevertheless, since all sub‐dimensions significantly correlate with contact quality—and given that the strongest association is found with overall multicultural ideology—these results suggest that the broader construct of multicultural ideology, encompassing all its sub‐dimensions, may contribute to positive interactions between the local population and migrants, as well as to harmonious intercultural relations.

This is particularly relevant in Italy, where “migrants” refers to individuals from diverse cultural backgrounds (OECD 2023). In this regard, multicultural ideology can function—like many ideological constructs—as a superordinate variable that shapes people's perceptions of reality, promoting positive intergroup relations.

Regarding political orientations, when analysing each sub‐dimension separately, CD emerges as the dimension most strongly related to right‐wing political orintations, showing negative associations. This suggests that individuals who perceive diversity as beneficial are less likely to support right‐wing ideologies, which often emphasise national identity, cultural homogeneity, and restrictive migration policies. This pattern is particularly relevant in the Italian political landscape, where political discourse surrounding migration has been highly polarised, with certain parties leveraging anti‐immigration rhetoric to shape public opinion (Prislei et al. 2022).

However, similar to the findings on intergroup contact, the broader multicultural ideology shows the strongest overall association with political orientation. This reinforces the idea that multicultural ideology is a superordinate framework that shapes individuals' perceptions of social and political realities.

While these studies offer important contributions, several limitations must be considered. First, data collection was conducted online, which may introduce sampling biases and limit the generalisability of the results. Future studies should aim to replicate these findings using more diverse sampling methods and representative samples, as well as by testing the model on additional samples.

Furthermore, although the study highlights the potential role of multicultural ideology in intergroup relations and political orientation, its cross‐sectional design prevents us from making any causal inferences. Additionally, further research is needed to examine the directionality of the proposed associations.

Despite its limitations, this research underscores the significant role of multicultural ideology in shaping intergroup relations and political attitudes in Italy. Adapting the MCI‐r scale provides a valuable tool for assessing multicultural attitudes in this cultural context and contributes to the broader field of cross‐cultural psychology.

## Ethics Statement

All procedures performed involving human participants were in accordance with the ethical standards of the [anonymised for review] and with the 1964 Helsinki Declaration and its later amendments.

## Consent

Informed consent was obtained from all individual adult participants included in the study.

## Conflicts of Interest

The authors declare no conflicts of interest.

## Endnotes

^1^
In addition, Stuart and Ward (2019) introduced the concept of normative multiculturalism, which refers to people's perceptions of how interactions between culturally diverse groups, multicultural policies and practices, and diversity‐valuing ideologies are viewed as everyday or normative aspects of their society.

^2^
However, it is essential to note that multicultural ideology differs significantly from other ideological frameworks, such as interculturalism and Social Dominance Orientation (SDO). Therefore, it cannot be assumed that these perspectives function similarly. Specifically, interculturalism distinguishes itself from multicultural ideology by being hierarchical, with one dominant culture holding a superior position within society. In contrast, multicultural ideology is non‐hierarchical, viewing all cultures as equal participants in a plural society (Grigoryev and Berry 2021). Similarly, SDO, which emphasises social hierarchies and group dominance, contrasts with multicultural ideology's inclusive and egalitarian stance. Research has shown that constructs like SDO, which influence individuals' perceptions of social hierarchies and intergroup relations, are strongly linked to multicultural ideology (Levin et al. 2012).

^3^
In the early stages of research, multicultural ideology was often assessed vaguely, typically involving only a few questions within broader surveys that measured various constructs across different societies (see Berry et al. 1977). However, researchers gradually moved toward more specific measures, often focusing on the acculturation preferences of local populations toward migrants as an indicator of multicultural ideology (see Berry et al. 1977).

^4^
Protocol number: UniCa—Prot. n. 0000610.

^5^
The AVE assess convergent validity, which refers to the proportion of variance in an indicator variable explained by the construct relative to the total variance of the indicators (Cheung and Wang 2017). The HTMT represents the average heterotrait‐heteromethod correlations (i.e., correlations between indicators measuring different constructs) relative to the average monotrait‐heteromethod correlations (i.e., correlations between indicators within the same construct; Cheung and Wang 2017).

^6^
We translated the 24 items and asked a bilingual researcher unfamiliar with the original scale to translate them back into the original language. Afterward, we made some adjustments to avoid awkwardness caused by literal translations and to ensure that the expressions made sense in Italian. Finally, the bilingual researcher performed another back‐translation of the revised items.

^7^
All the analyses performed have been shared on: https://osf.io/kmjdq/?view_only=9c6979b915374e3a931edd2fbe0e77b9.

^8^
Indicators of model adequacy were set at TLI > 0.90, CFI > 0.95, RMSEA ≤ 0.06, and SRMR ≤ 0.08 (see Kline 2016). It is important to note that fit indices like TLI and CFI are sensitive to model complexity and that cutoffs are not definitive indicators of model validity (Kline 2016).

^9^
The thresholds were established based on Cheung and Rensvold (2002), with changes in ΔCFI ≤ 0.01 indicating that models are comparable.

^10^
The AVE values higher than 0.50 indicate convergent validity and the HTMT values lower than 0.85 are considered supportive of discriminant validity (Cheung and Wang 2017).

^11^
The factor loadings for the four‐factor solution are reported in Table A2 (see Appendix).

^12^
For configural invariance, the fit indices suggested that the factor structure was invariant between women and men, meaning that the same factor structure holds across groups. Similarly, the indices for metric invariance and scalar invariance indicated that the strength of the relationship between observed variables and latent factors is comparable as well as factor means. Therefore, we can accept the more restrictive model without any issues.

^13^
To further validate our findings, we performed a CFA on the best‐fitting model identified in Study 1, specifically examining both the four‐factor solution and the hierarchical model. We employed the same model fit indices as in Study 1 to evaluate the models and compare the two solutions (i.e., four‐factor and hierarchical one). The CFA results indicated good fit indices for both models. The four‐factor solution χ^2^(98) = 172.36, *p* < 0.001, CFI = 0.911, TLI = 0.891, RMSEA = 0.066, 90% CI [0.049, 0.082], SRMR = 0.059, and the hierarchical model χ^2^(100) = 177.383, *p* < 0.001, CFI = 0.907, TLI = 0.888, RMSEA = 0.062, 90% CI [0.050, 0.082], SRMR = 0.061. The slight difference in ΔCFI = 0.002 confirmed that both solutions are equally viable, consistent with the findings from Study 1. Based on these results, the subsequent analyses will incorporate the four‐factor dimensions and a composite measure of Multicultural Ideology, which treats each of the four subscales as single‐item indicators.

^14^
We employed the same measure as Lefringhausen et al. (2024).

^15^
All the analyses performed have been shared on: https://osf.io/kmjdq/?view_only=9c6979b915374e3a931edd2fbe0e77b9.

## Appendix A

**TABLE A1:** Italian adaptation of the revised multicultural ideology scale.

	English	Italian
	In the following, we will ask you questions about your opinion about multiculturalism in Italy. Please read the following statements and indicate to what extent you agree or disagree. Some of these statements are Worded very similarly, differing only by a few words or formulation. Thus, to allow us to capture your opinions as accurate as possible, please ensure to read each statement very carefully. If you prefer not to indicate an answer to any of these statements, then just move forward in the survey.	Successivamente, ti faremo delle domande in merito alle tue opinioni sul multiculturalismo in Italia. Per favore, leggi le seguenti affermazioni e indica in che grado sei d'accordo o in disaccordo (1 = totalmente in disaccordo, 2 = un po' in disaccordo, 3 = né d'accordo né in disaccordo, 4 = un po' d'accordo, 5 = totalmente d'accordo). Alcune delle affermazioni sono formulate in maniera simile e differiscono solo di alcune parole. Per permetterci di rilevare le tue opinioni in maniera più accurata possibile, per favore assicurati di leggere ogni affermazione accuratamente. Se preferisci non rispondere a qualche domanda, puoi lasciarla e passare a quella successiva.
Cultural Maintenance (CM)	It would be good to see all ethnic groups in Italy retain their cultures.	Sarebbe bello vedere tutti i gruppi etnici presenti in Italia mantenere le loro culture d'origine.
	It is best for Italy if all ethnic groups forgot their cultural backgrounds as soon as possible. [Reverse]	Sarebbe meglio per l'Italia se tutti i gruppi etnici qui presenti dimenticassero il loro background culturale il prima possibile. [Reverse]
	People who come to Italy should change their behaviour to be more similar to the local population. [Reverse]	Le persone che vengono in Italia dovrebbero cambiare i loro comportamenti in modo che siano più simili a quelli della popolazione locale. [Reverse]
	We should help ethnic and racial minorities preserve their cultural heritages in Italy.	Dovremmo aiutare le minoranze etniche a preservare le loro origini culturali in Italia.
Equity/Inclusion (EQ)	I think that immigrants in Italy should have equal rights as people already living here.	Penso che i migranti in Italia dovrebbero avere gli stessi diritti delle persone che già vivono qui.
	I think that immigrants in Italy should have fewer rights than those who were born here. [Reverse]	Penso che i migranti in Italia dovrebbero avere meno diritti rispetto a coloro che sono nati qui. [Reverse]
	I think that immigrants in Italy should enjoy the same freedoms as those who were born here.	Penso che i migranti in Italia dovrebbero godere delle stesse libertà di coloro che sono nati qui.
	I think that immigrants in Italy should not have the same freedoms as those already living here. [Reverse]	Penso che i migranti in Italia non dovrebbero avere le stesse libertà di coloro che già vivono qui. [Reverse]
Social Interaction (SI)	If members of ethnic groups want to keep their own culture, they should keep it to themselves and not bother other people in this country. [Reverse]	Se i membri dei gruppi etnici volessero mantenere la propria cultura, dovrebbero tenerla per sé, senza disturbare le persone di questo paese. [Reverse]
	We should do more to learn about the customs and heritage of different ethnic and cultural groups in this country.	Dovremmo imparare di più sui costumi e le tradizioni dei gruppi etnici e culturali presenti nel nostro paese.
	I do not like being on a bus or a train in which there are many people of other cultures. [Reverse]	Non mi piace stare su un autobus o un treno nel quale ci sono tante persone di altre culture. [Reverse]
	I think that immigrants and people in Italy should seek more contact with one another.	Penso che i migranti e gli italiani dovrebbero entrare maggiormente in contatto gli uni con gli altri.
Essentialistic Boundaries (EB)	Ethnic and cultural group categories are not very important, and so should not be used for understanding about other people.	Le categorie di gruppi etnici e culturali non sono molto importanti e quindi non dovrebbero essere utilizzate per comprendere le altre persone.
	Racial and ethnic group memberships do not matter very much to who we really are.	Appartenere a gruppi etnici e culturali non è importante per chi siamo.
	All human beings are individuals, and therefore race and ethnicity are not important.	Tutti gli esseri umani sono persone, e perciò la provenienza etnica e culturale non hanno importanza.
	At our core, all human beings are really all the same, so racial and ethnic categories do not matter.	Alla fine, tutti gli esseri umani sono uguali, per cui le categorie etniche e culturali non importano.
Extent of Differences (DI)	All cultures should have their own distinct traditions and perspectives.	Tutte le culture dovrebbero avere le proprie distinte tradizioni e prospettive.
	Each racial and ethnic group should have their own distinguishing characteristics.	Ogni gruppo etnico e culturale dovrebbe avere le proprie caratteristiche distinguibili
	All groups in Italy should clearly show their own distinctive cultural features.	Tutti i gruppi in Italia dovrebbero mostrare chiaramente le proprie caratteristiche culturali distinte.
	It is important for all groups in Italy to keep themselves distinct from other groups.	È importante per tutti i gruppi in Italia restare distinti dagli altri gruppi.
Consequences of diversity (CD)	Having a lot of different cultural groups in Italy makes it difficult to solve problems in our society. [Reverse]	Avere molti gruppi culturali diversi in Italia rende difficile risolvere i problemi della nostra società. [Reverse]
	The unity of this country is weakened by ethnic groups sticking to their old ways. [Reverse]	L'unità di questo paese è indebolita dai gruppi etnici che restano attaccati ai loro modi di fare. [Reverse]
	A society which has a variety of ethnic groups is more able to tackle new problems as they occur.	Una società con una varietà culturale è più capace di risolvere problemi che le accadono.
	I feel at ease when I am in a city with many immigrants.	Mi sento a mio agio quando sono in una città con tanti migranti.

**TABLE A2:** Factor loadings of the italian adaptation of the revised multicultural ideology scale.

	Items	Loadings scaled
Cultural Maintenance (CM)	It would be good to see all ethnic groups in Italy retain their cultures.	0.67
	It is best for Italy if all ethnic groups forgot their cultural backgrounds as soon as possible. [Reverse]	−0.76
	People who come to Italy should change their behaviour to be more similar to the local population. [Reverse]	−0.67
	We should help ethnic and racial minorities preserve their cultural heritages in Italy.	0.76
Equity/Inclusion (EQ)	I think that immigrants in Italy should have equal rights as people already living here.	0.86
	I think that immigrants in Italy should have fewer rights than those who were born here. [Reverse]	−0.83
	I think that immigrants in Italy should enjoy the same freedoms as those who were born here.	0.79
	I think that immigrants in Italy should not have the same freedoms as those already living here. [Reverse]	−0.57
Social Interaction (SI)	If members of ethnic groups want to keep their own culture, they should keep it to themselves and not bother other people in this country. [Reverse]	0.62
	We should do more to learn about the customs and heritage of different ethnic and cultural groups in this country.	−0.64
	I do not like being on a bus or a train in which there are many people of other cultures. [Reverse]	0.65
	I think that immigrants and people in Italy should seek more contact with one another.	−0.66
Consequences of diversity (CD)	Having a lot of different cultural groups in Italy makes it difficult to solve problems in our society. [Reverse]	0.68
	The unity of this country is weakened by ethnic groups sticking to their old ways. [Reverse]	0.77
	A society which has a variety of ethnic groups is more able to tackle new problems as they occur.	−0.67
	I feel at ease when I am in a city with many immigrants.	−0.63
